# A Study of the Prevalence of Diabetic Retinopathy in Patients With Ischemic Heart Disease and Diabetes Mellitus

**DOI:** 10.7759/cureus.65005

**Published:** 2024-07-20

**Authors:** Ozukhil Radhakrishnan, Khushboo Goyal, Vishakha Vatkar, Shreya Gandhi, Tushar Agrawal

**Affiliations:** 1 Ophthalmology, Cornea, Glaucoma, Dr. D.Y. Patil Medical College, Hospital and Research Centre, Pune, IND; 2 Ophthalmology, Dr. D.Y. Patil Medical College, Hospital and Research Centre, Pune, IND

**Keywords:** ejection fraction, macrovascular, microvascular, ischemic heart disease, diabetes mellitus, diabetic retinopathy

## Abstract

Background

Diabetes mellitus is one of the most important and common chronic diseases worldwide and is expected to increase in prevalence. Diabetic retinopathy (DR) is one of the most prevalent microvascular sequelae of diabetes mellitus (DM), and ischemic heart disease is a macrovascular sequela. This study was conducted to find out the relation between the degree of DR and ischemic heart disease severity in Indian patients.

Materials and methods

This cross-sectional, descriptive, hospital-based study was conducted in the ophthalmology department at Dr. D. Y. Patil Medical Hospital, Pune, Maharashtra, India, from September 2022 to June 2024. A total of 200 eyes from 100 patients who were diagnosed with cases of ischemic heart disease and diabetes mellitus were included in the study. Patients with corneal pathology like endothelial dystrophies, corneal degenerations, corneal scars, or trauma preventing good visualization of the posterior segment were excluded from the study. Patients with active uveitis, patients with a history of undergoing any previous vitreoretinal surgery or laser procedures, non-compliant patients, patients not willing to undergo the procedure, or those not consenting to the study were also excluded. Written informed consent was obtained from each patient. Data was entered in Microsoft Excel and statistical analysis was done using IBM Corp. Released 2019. IBM SPSS Statistics for Windows, Version 26.0. Armonk, NY: IBM Corp. As the continuous variables showed a skewed distribution, we used the Mann-Whitney test and the Kruskal-Wallis test to test the significance of the difference between continuous and categorical variables. A chi-square test was employed to check the association between categorical variables. Significance was assumed at an alpha error of 5%.

Results

The prevalence of diabetic retinopathy was found to be 95%. The mean age of patients with DR and patients with no diabetic retinopathy was 58.38 and 59.40 years, respectively, with the majority of the patients being in the age group of 60-69 years (46%). The majority of the patients were males (65%), while 35% were females. There was a significant association between the severity of diabetic retinopathy and the higher HbA1c levels, the use of insulin as a treatment modality, and the higher blood sugar levels in our study population. It was observed that the patients in our study with an ejection fraction of <40% had significantly higher severity of diabetic retinopathy in the form of PDR and high-risk PDR. The severity of the DR was directly correlated with the severity of IHD in our study, with most of the IHD patients with a 40-60% ejection fraction having moderate NPDR and patients with a >60% ejection fraction having mild or moderate NPDR.

Conclusion

The prevalence of diabetic retinopathy among the IHD patients with diabetes was 95% in our study, with moderate NPDR being the most common stage of DR seen among the patients. It was observed that more severe stages of diabetic retinopathy were seen in patients who were on treatment with insulin than in patients who were on treatment with OHA. Severe stages of diabetic retinopathy were associated with higher blood sugar levels (BSL) and higher glycated hemoglobin levels. In the present study, it was observed that a lower ejection fraction (<40%), which is a marker of reduced cardiac function, was associated with more severe stages of diabetic retinopathy.

## Introduction

Diabetes mellitus (DM) is one of the most important and common chronic diseases worldwide and is expected to increase in prevalence. The prevention of DM complications is important because morbidity, mortality, and healthcare costs for diabetic patients are critical socioeconomic issues in most countries [1]. Diabetes mellitus (DM) is a vascular disease that has several microvascular manifestations, such as retinopathy, and macrovascular complications, such as coronary artery disease. Type 2 DM has been identified as an independent risk factor for peripheral arterial disease, congestive heart failure (CHF), and cardiovascular disease [2].

Many diabetics who have retinopathy may also have undiagnosed coronary artery disease. One of the well-known macrovascular consequences of diabetes mellitus is coronary heart disease (CHD), which is a major cause of mortality in individuals with the condition [3]. Diabetic retinopathy (DR) is the most common cause of visual disability in people of working age [4]. It is well known that the risk factors for DR include the duration of DM, hypertension, poor glycemic control, and obesity. With a worldwide prevalence of 382 million, diabetic retinopathy (DR) is one of the most prevalent diabetes-specific sequelae [4,5]. DR is a rapidly growing condition that may cause damage to the retinal capillaries and ultimately result in blindness.

The importance of glycemic control in reducing microvascular complications is well established. Although controversial, there is evidence that glycemic control can limit macrovascular disease, including cardiovascular disease, stroke, and peripheral arterial disease [6,7]. Hemodynamics (impaired autoregulation and hyperperfusion) and vascular endothelial growth factor (VEGF) are the possible mechanisms by which hypertension may affect DR [8]. Extensive data linking DR to various micro- and macrovascular problems is available. It was recently shown that DR is linked to macrovascular comorbidities such as coronary disease and cerebrovascular accidents, as well as subclinical atherosclerosis [9].

The purpose of the study is to find out the prevalence of diabetic retinopathy in patients with ischemic heart disease (IHD), to know if more severe stages of diabetic retinopathy are associated with an increased occurrence of IHD, and to find out the correlation between macrovascular and microvascular complications of diabetes mellitus. There are no studies from this part of the country that study this aspect of diabetic retinopathy to the best of our knowledge.

Aim and objectives

The study aims to study the prevalence of diabetic retinopathy in patients with ischemic heart disease and diabetes mellitus and find out the relation between the degree of diabetic retinopathy and ischemic heart disease severity in Indian patients.

## Materials and methods

Study design and setting

This cross-sectional, descriptive, hospital-based study was conducted in the ophthalmology department at Dr. D. Y. Patil Medical Hospital, Pune, Maharashtra, India, from September 2022 to June 2024. A total of 200 eyes of 100 patients who were diagnosed with cases of ischemic heart disease and diabetes mellitus were included in the study, with each participant undergoing a thorough clinical assessment and investigation. The study received approval from the Institutional Ethics Committee of DYPMCH, Pune (approval number IESC/PGS/2022/112, dated January 28, 2022).

Inclusion criteria

All patients who were diagnosed with cases of ischemic heart disease and type 2 diabetes mellitus in the age group of 40-70 years at a tertiary care center in Western Maharashtra were included in the study.

Exclusion criteria

Patients with corneal pathology like endothelial dystrophies or corneal degenerations, corneal scars, dense cataracts, or trauma preventing good visualization of the fundus were excluded from the study. Also excluded were patients with active uveitis, patients with a history of undergoing any previous vitreoretinal surgery or laser procedures, non-compliant patients, and patients not willing to undergo the procedure or not consenting to the study.

Sample size

Based on a study conducted by El Demerdash et al. [3], the minimum sample size was calculated to be 100 using WinPepi software, version 11.38.

Data and sample collection

All patients diagnosed with IHD and DM were subjected to the following: History taking, including age, sex, socioeconomic status, employment status, smoking history (current, former, or never), duration of DM, and family history of IHD, DM, and HTN.

Thorough clinical examination: Laboratory investigation, including complete blood count (CBC), HbA1C, blood sugar levels, serum total cholesterol, CK-MB, troponin-I, NT-pro BNP, serum creatinine, and total cholesterol (TC). To correlate clinically, 2D-echo and ECG were performed for all the patients. A thorough ophthalmic evaluation including uncorrected visual acuity (UCVA) and best-corrected visual acuity (BCVA) for both near and distance vision was measured using the Snellen chart; ocular examination of the orbit and adnexa and extraocular movements were assessed; anterior segment examination with slit lamp biomicroscopy was conducted to assess the conjunctiva, cornea, anterior chamber, iris details, pupil, and lens; and posterior segment evaluation was done using slit lamp biomicroscopy using a 90D Volk lens and an indirect ophthalmoscope with a 20D lens. (The patient's eye was dilated using 0.8% tetracycline and 5% phenylephrine.).

Intraocular pressure was measured in all patients using non-contact air-puff tonometry. Diabetic retinopathy was graded using the early treatment diabetic retinopathy study (ETDRS) system. Optical coherence tomography and fundus fluorescein angiography were done for posterior segment evaluation when indicated.

Consent

Written informed consent was obtained from each patient. In the study, we recruited 100 patients, explained the procedure and purpose to them, and obtained informed consent from all the patients.

Statistical analysis

Data was entered in Microsoft Excel and statistical analysis was done using IBM Corp. Released 2019. IBM SPSS Statistics for Windows, Version 26.0. Armonk, NY: IBM Corp. Quantitative data was summarized using the mean (SD) and median (IQR). Qualitative data was summarized using proportions. As the continuous variables showed a skewed distribution, we used the Mann-Whitney test and the Kruskal-Wallis test to test the significance of the difference between continuous and categorical variables. A chi-square test was employed to check the association between categorical variables. Significance was assumed at an alpha error of 5%.

## Results

**Figure 1:**
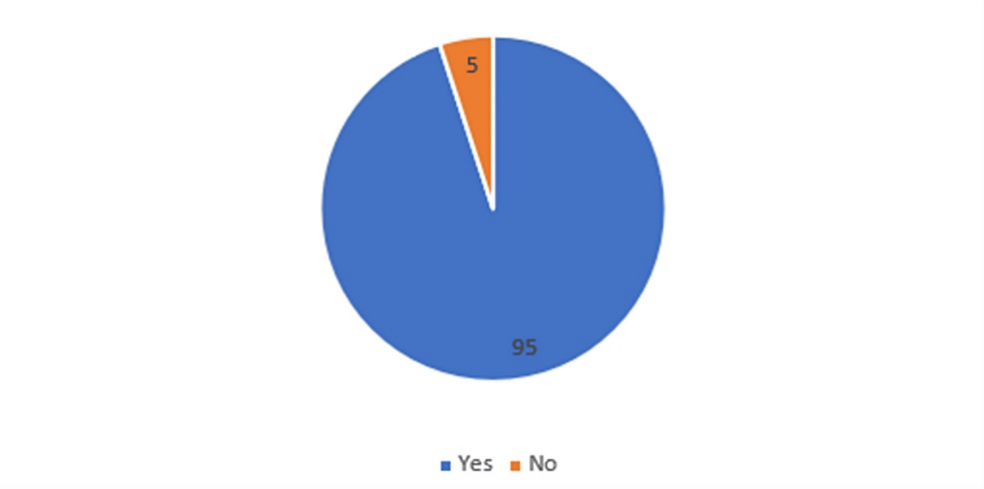
Diabetic retinopathy prevalence

Table 1, Figure 1 show the prevalence of diabetic retinopathy in the study population. The prevalence of diabetic retinopathy among the IHD patients was 95%.

**Table 1:** Prevalence of diabetic retinopathy in the study population

Yes/No	Frequency	Percent
Yes	95	95.0
No	5	5.0
Total	100	100.0

**Figure 2:**
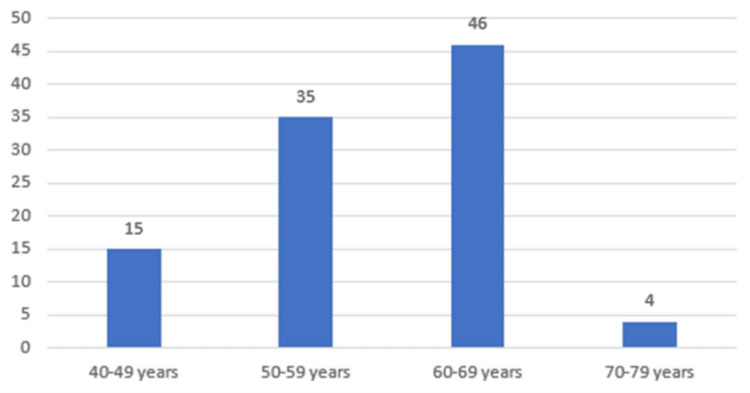
Age distribution of patients

Table 2, Figure 2 show the age distribution in the study population. The majority of the patients in our study were in the age group of 60-69 years (46%), followed by 50-59 years (35%), and 40-49 years (15%).

**Table 2:** Age distribution of patients in the study population

Age group	Frequency	Percent
40-49 years	15	15.0
50-59 years	35	35.0
60-69 years	46	46.0
70-79 years	4	4.0
Total	100	100.0

**Figure 3:**
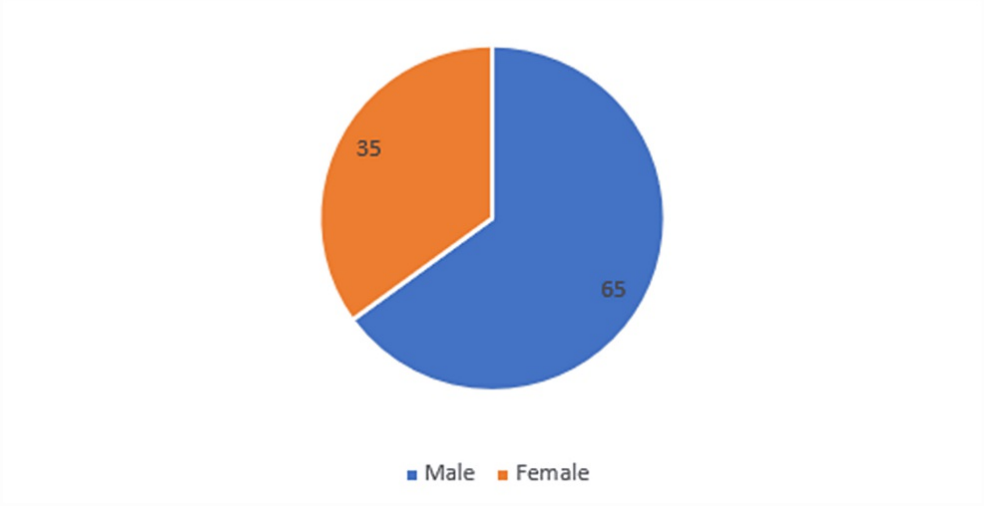
Gender distribution of patients

Table 3, Figure 3 show the gender distribution in the study population. Most of the patients were males (65%), while 35% of them were females.

**Table 3:** Gender distribution of patients in the study population

Gender	Frequency	Percent
Male	65	65.0
Female	35	35.0
Total	100	100.0

**Figure 4:**
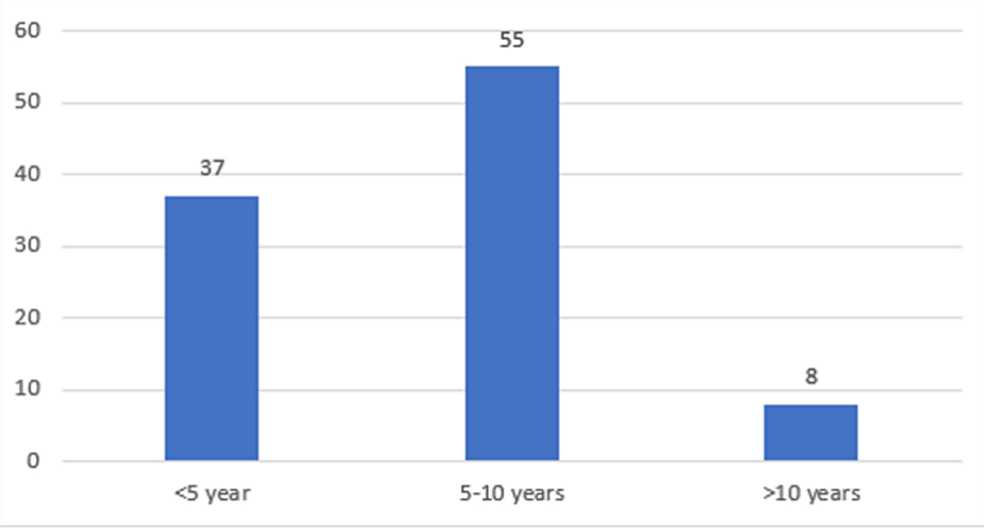
Duration of DM

Table 4, Figure 4 show the duration of DM in the study population. Most of the patients had diabetes for 5-10 years (55%), while 37% had DM for less than five years and 8% had it for more than 10 years.

**Table 4:** Duration of DM in the study population

Age range	Frequency	Percent
<5 year	37	37.0
5-10 years	55	55.0
>10 years	8	8.0
Total	100	100.0

**Figure 5:**
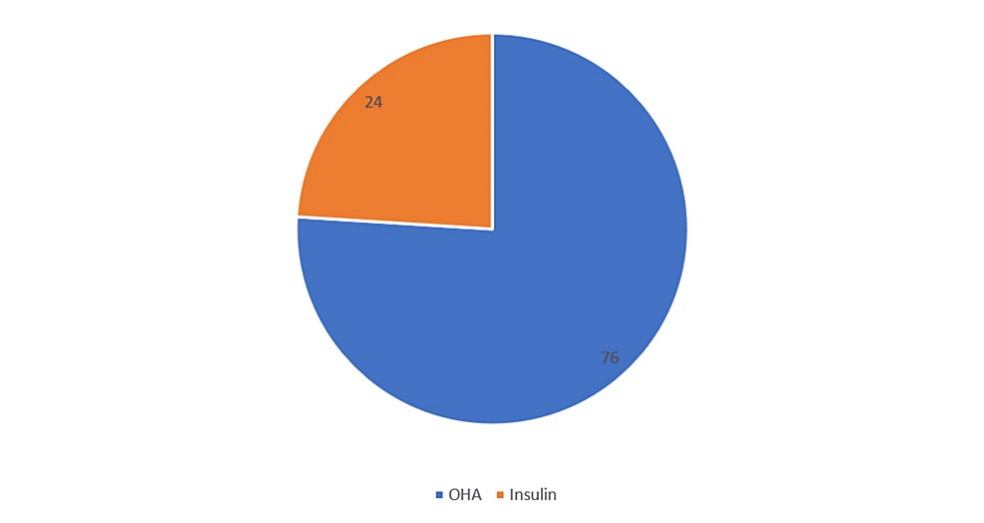
Treatment modality of DM

Table 5, Figure 5 shows the treatment modality for DM in the study population. Most of the diabetic patients were on treatment with OHA (76%), while 24% were on treatment with insulin.

**Table 5:** Treatment distribution of DM in the study population

OHA/Insulin	Frequency	Percent
OHA	76	76.0
Insulin	24	24.0
Total	100	100.0

**Figure 6:**
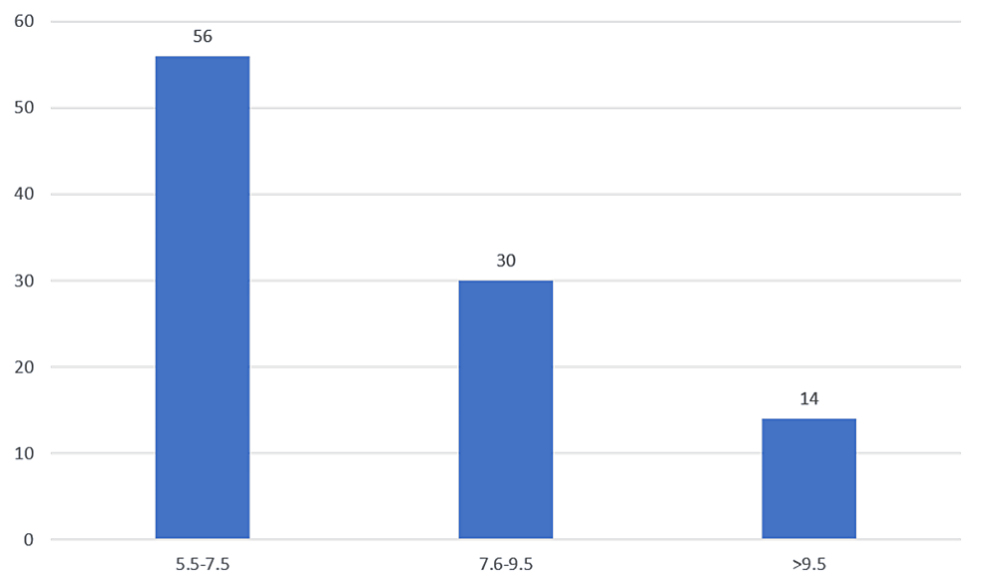
HbA1c levels

Table 6, Figure 6 show the glycated hemoglobin values in the study population. Most of the patients had HbA1c values of 5.5-7.5 (56%), followed by HbA1c values of 7.6-9.5 (30%) and >9.5 (14%).

**Table 6:** Glycated hemoglobin (HbA1c) levels of the study population

Range	Frequency	Percent
5.5-7.5	56	56.0
7.6-9.5	30	30.0
>9.5	14	14.0
Total	100	100.0

**Figure 7:**
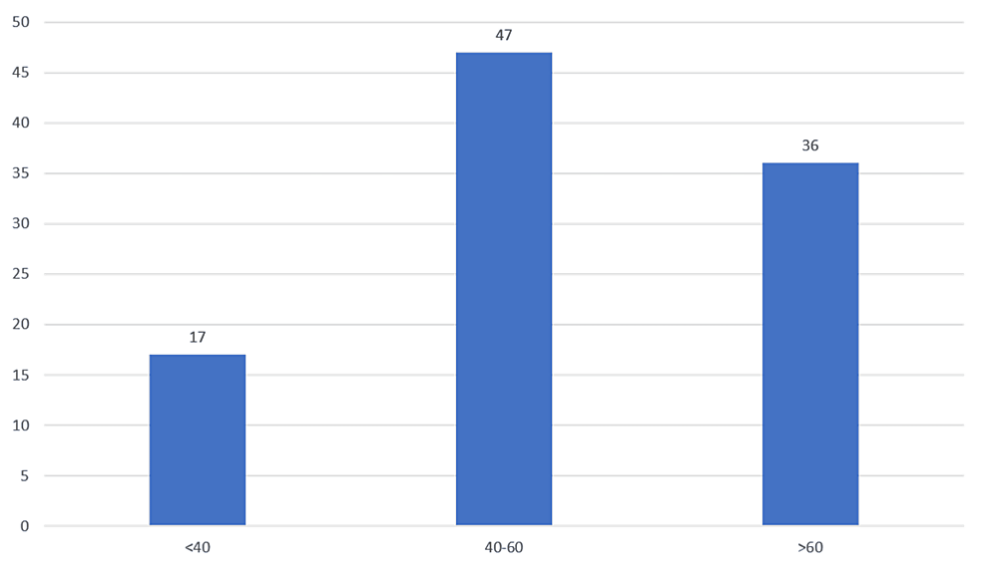
Ejection fraction values

Table 7, Figure 7 show the ejection fraction values obtained from 2D-echo in the study population. According to 2D-echo, most of the patients had an ejection fraction of 40-60% (47%), followed by a value of >60% (36%) and a value of <40% (17%).

**Table 7:** Ejection fraction values of the study population

Range	Frequency	Percent
<40	17	17.0
40-60	47	47.0
>60	36	36.0
Total	100	100.0

Table 8 shows the correlation of diabetic retinopathy with age, duration of IHD and DM, troponin I, CKMB, FLP, BSL (R), and HbA1c (descriptive data).

**Table 8:** Correlation of diabetic retinopathy with age, duration of IHD and DM, Troponin I, CKMB, FLP, BSL(R), and HbA1c values in the study population IHD: Ischemic heart disease, DM: Diabetes mellitus, CKMB: Creatinine Kinase-MB, BSL(R): Blood sugar levels-random, HbA1c: Glycated hemoglobin

	Diabetic Retinopathy Yes				Diabetic Retinopathy No			
	Mean	SD	Median	IQR	Mean	SD	Median	IQR
Age	58.38	59.00	7.45	53,64	59.40	62.00	10.78	49,68.5
Duration IHD	4.40	4.00	2.64	3,6	5.40	2.00	8.20	1,11.5
Duration DM	5.97	6.00	3.65	3,8	3.40	2.00	1.95	2,5.5
Duration HTN	5.14	5.00	3.43	3,6	1.67	2.00	0.58	1,
Troponin I (ng/mL)	0.28	0.24	0.13	0.18,0.34	0.15	0.14	0.05	0.115,0.2
CKMB (IU/L)	25.45	25.00	6.70	22,31	16.40	18.00	6.11	11.5,20.5
FLP	233.83	231.00	30.48	212,256	205.40	186.00	37.56	180,240.5
BSL (R) mg/dl	239.36	231.00	45.38	210,275	159.20	168.00	37.05	120.5,193.5
HbA1c (%)	7.88	7.40	1.72	6.8,8.4	5.90	5.60	0.68	5.5,6.45

The mean age of the patients with diabetic retinopathy and the patients without diabetic retinopathy was 58.38 and 59.40 years, respectively.

The mean duration of IHD in patients with diabetic retinopathy and in patients without diabetic retinopathy was 4.4 and 5.4 years, respectively.

The mean duration of diabetes for patients with diabetic retinopathy and patients without diabetic retinopathy was 5.97 and 3.4 years, respectively.

The mean duration of hypertension in patients with diabetic retinopathy and in patients without diabetic retinopathy was 5.14 and 1.67 years, respectively.

The mean CKMB of the patients with diabetic retinopathy and patients with no diabetic retinopathy was 25.45 and 16.40 IU/L, respectively.

The mean fasting lipid profile (FLP) values of the patients with diabetic retinopathy and patients without diabetic retinopathy were 233.83 and 205.4, respectively.

The mean BSL of the patients with diabetic retinopathy and patients with no diabetic retinopathy was 239.36 and 159.2 mg/dl, respectively.

The mean HbA1c of the patients with diabetic retinopathy and patients with no diabetic retinopathy was 7.88 and 5.9, respectively.

Table 9 shows that the duration of hypertension (p-value 0.020), CKMB (p-value 0.005), Troponin I (p-value 0.007), FLP (p-value 0.049), FBS (p-value 0.001), and HbA1c (p-value 0.002) were significantly higher among the diabetic retinopathy group than the non-DR group, as derived by the Mann-Whitney Test.

**Table 9:** Correlation of diabetic retinopathy with age, duration of IHD and DM, Troponin I, CKMB, FLP, BSL(R), and HbA1c values in the study population as derived by the Mann-Whitney Test IHD: Ischemic heart disease, DM: Diabetes mellitus, CKMB: Creatinine Kinase-MB, BSL(R): Blood sugar levels-random, HbA1c: Glycated hemoglobin, FLP: Fasting lipid profile

	Diabetic retinopathy	N	Mean rank	Sum of ranks	p-value
Age	Yes	95	50.15	4764.50	0.601
	No	5	57.10	285.50	
	Total	100			
Duration IHD	Yes	95	51.42	4885.00	0.162
	No	5	33.00	165.00	
	Total	100			
Duration DM	Yes	95	51.67	4909.00	0.076
	No	5	28.20	141.00	
	Total	100			
Duration HTN	Yes	35	20.71	725.00	0.020
	No	3	5.33	16.00	
	Total	38			
Troponin I (ng/mL)	Yes	94	51.79	4868.50	0.007
	No	5	16.30	81.50	
	Total	99			
CKMB (IU/L)	Yes	95	52.35	4973.00	0.005
	No	5	15.40	77.00	
	Total	100			
FLP	Yes	95	51.81	4922.00	0.049
	No	5	25.60	128.00	
	Total	100			
BSL (R) mg/dl	Yes	95	52.67	5004.00	0.001
	No	5	9.20	46.00	
	Total	100			
HbA1c (%)	Yes	95	52.57	4994.50	0.002
	No	5	11.10	55.50	
	Total	100			

**Figure 8:**
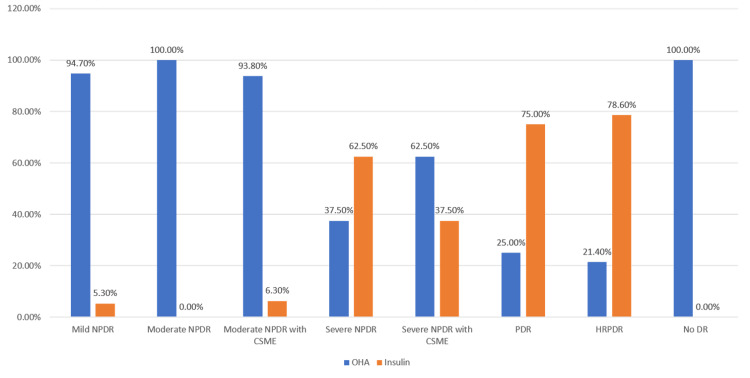
Correlation between treatment modality and DR severity DR: Diabetic retinopathy

Table 10, Figure 8 show the correlation of the severity of diabetic retinopathy with treatment of DM with OHA and insulin as derived by the Kruskal-Wallis test. A higher proportion of the severe forms of DR patients were on treatment with insulin than the milder forms of DR (p-value <0.001).

**Table 10:** Correlation of severity of diabetic retinopathy with treatment of DM with OHA and insulin DR: Diabetic retinopathy, OHA: Oral hypoglycemic agent, HRPDR: High-risk proliferative diabetic retinopathy, PDR: Proliferative diabetic retinopathy, NPDR: Non-proliferative diabetic retinopathy, CSME: Clinically significant macular edema

			Treatment		p-value
			OHA	Insulin	
DR severity	Mild NPDR	Frequency	18	1	<0.001
		Percentage	94.7%	5.3%	
	Moderate NPDR	Frequency	26	0	
		Percentage	100.0%	0.0%	
	Moderate NPDR with CSME	Frequency	15	1	
		Percentage	93.8%	6.3%	
	Severe NPDR	Frequency	3	5	
		Percentage	37.5%	62.5%	
	Severe NPDR with CSME	Frequency	5	3	
		Percentage	62.5%	37.5%	
	PDR	Frequency	1	3	
		Percentage	25.0%	75.0%	
	HRPDR	Frequency	3	11	
		Percentage	21.4%	78.6%	
	No DR	Frequency	5	0	
		Percentage	100.0%	0.0%	
Total		Frequency	76	24	
		Percentage	76.0%	24.0%	

Table 11 shows the correlation of the severity of diabetic retinopathy with the blood sugar levels as derived by the Kruskal-Wallis test. There is a significant association between the severity of diabetic retinopathy and higher blood sugar levels (p-value <0.001). 

**Table 11:** Correlation of the severity of diabetic retinopathy with blood sugar levels in the study population BSL (R): Blood sugar levels random, DR: Diabetic retinopathy, OHA: Oral hypoglycemic agent, HRPDR: High-risk proliferative diabetic retinopathy, PDR: Proliferative diabetic retinopathy, NPDR: Non-proliferative diabetic retinopathy, CSME: Clinically significant macular edema

	DR severity	N	Mean rank	p-value
BSL (R) mg/dl	No DR	5	9.20	<0.001
	Mild NPDR	19	23.68	
	Moderate NPDR	26	40.37	
	Moderate NPDR with CSME	16	45.66	
	Severe NPDR	8	76.56	
	Severe NPDR with CSME	8	72.31	
	PDR	4	96.13	
	HRPDR	14	85.61	
	Total	100		

Table 12 shows the correlation of the severity of diabetic retinopathy with the glycated hemoglobin levels as derived by the Kruskal-Wallis test. There is a significant association between the severity of diabetic retinopathy and the higher HbA1c levels (p-value <0.001).

**Table 12:** Correlation of severity of diabetic retinopathy with glycated hemoglobin levels in the study population DR: Diabetic retinopathy, HRPDR: High-risk proliferative diabetic retinopathy, PDR: Proliferative diabetic retinopathy, NPDR: Non-proliferative diabetic retinopathy, CSME: Clinically significant macular edema, HbA1c: Hemoglobin A1C

	DR severity	N	Mean Rank	p-value
HbA1c (%)	No DR	5	11.10	<0.001
	Mild NPDR	19	26.00	
	Moderate NPDR	26	42.75	
	Moderate NPDR with CSME	16	46.66	
	Severe NPDR	8	56.75	
	Severe NPDR with CSME	8	74.81	
	PDR	4	93.50	
	HRPDR	14	86.86	
	Total	100		

**Figure 9:**
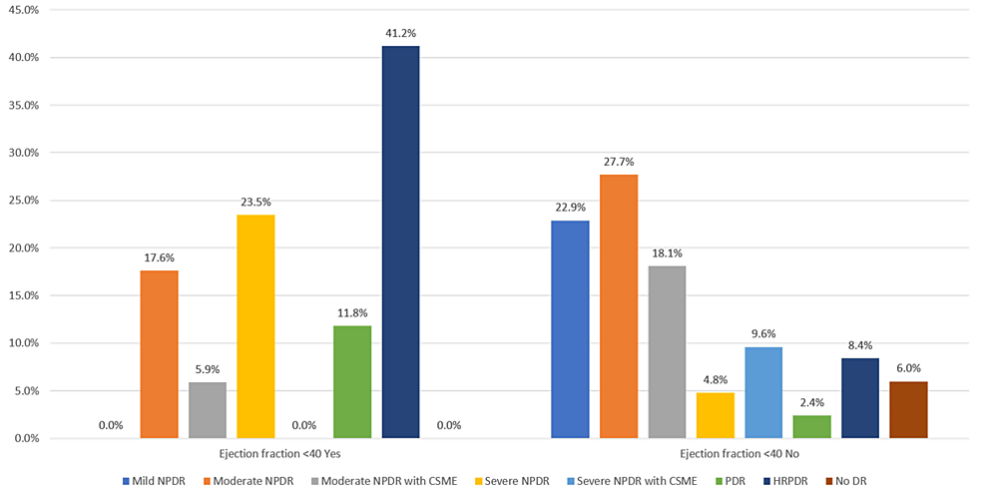
Correlation between ejection fraction <40 and diabetic retinopathy severity

Table 13, Figure 9 show the correlation of the severity of diabetic retinopathy with an ejection fraction <40 as derived by the Kruskal-Wallis test. A significantly higher proportion of patients with an ejection fraction of <40 had PDR and HRPDR (p-value <0.001).

**Table 13:** Correlation of severity of diabetic retinopathy with ejection fraction <40 NPDR: Non-proliferative diabetic retinopathy, CSME: Clinically significant macular edema, DR: Diabetic retinopathy, HRPDR: High-risk proliferative diabetic retinopathy

Correlation of severity of diabetic retinopathy with ejection fraction <40					
			Ejection fraction <40		p-value
			Yes	No	
DR severity	Mild NPDR	Frequency	0	19	<0.001
		Percentage	0.0%	22.9%	
	Moderate NPDR	Frequency	3	23	
		Percentage	17.6%	27.7%	
	Moderate NPDR with CSME	Frequency	1	15	
		Percentage	5.9%	18.1%	
	Severe NPDR	Frequency	4	4	
		Percentage	23.5%	4.8%	
	Severe NPDR with CSME	Frequency	0	8	
		Percentage	0.0%	9.6%	
	PDR	Frequency	2	2	
		Percentage	11.8%	2.4%	
	HRPDR	Frequency	7	7	
		Percentage	41.2%	8.4%	
	No DR	Frequency	0	5	
		Percentage	0.0%	6.0%	
Total		Frequency	17	83	
		Percentage	100.0%	100.0%	

**Figure 10:**
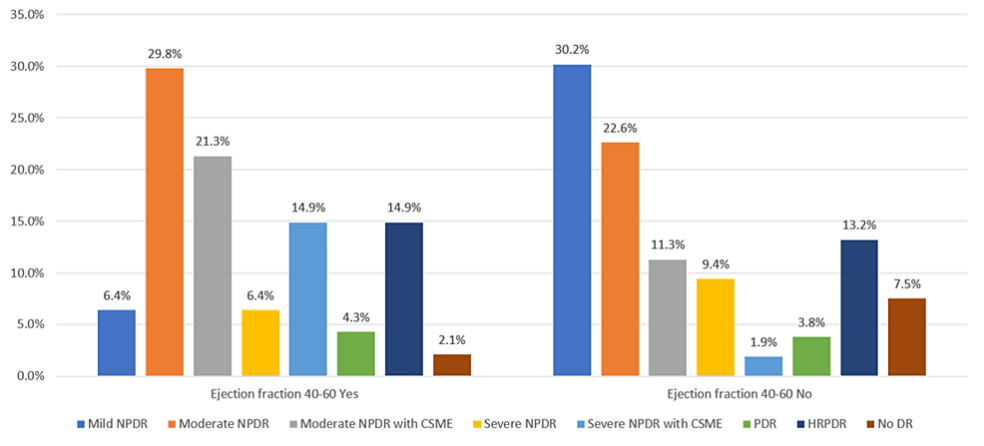
Correlation between ejection fraction 40-60 and diabetic retinopathy severity

Table 14, Figure 10 show the correlation of the severity of diabetic retinopathy with ejection fraction 40-60 as derived by the Kruskal-Wallis test. A significantly higher proportion of patients with an ejection fraction of 40-60 had moderate NPDR (p-value 0.015).

**Table 14:** Correlation of severity of diabetic retinopathy with ejection fraction 40-60 DR: Diabetic retinopathy, HRPDR: High-risk proliferative diabetic retinopathy, PDR: Proliferative diabetic retinopathy, NPDR: Non-proliferative diabetic retinopathy, CSME: Clinically significant macular edema

			Ejection fraction 40-60		p-value
			Yes	No	
DR severity	Mild NPDR	Frequency	3	16	0.015
		Percentage	6.4%	30.2%	
	Moderate NPDR	Frequency	14	12	
		Percentage	29.8%	22.6%	
	Moderate NPDR with CSME	Frequency	10	6	
		Percentage	21.3%	11.3%	
	Severe NPDR	Frequency	3	5	
		Percentage	6.4%	9.4%	
	Severe NPDR with CSME	Frequency	7	1	
		Percentage	14.9%	1.9%	
	PDR	Frequency	2	2	
		Percentage	4.3%	3.8%	
	HRPDR	Frequency	7	7	
		Percentage	14.9%	13.2%	
	No DR	Frequency	1	4	
		Percentage	2.1%	7.5%	
Total		Frequency	47	53	
		Percentage	100.0%	100.0%	

Table 15 shows the correlation between the severity of diabetic retinopathy and ejection fraction >60 as derived by the Kruskal-Wallis test. A significantly higher proportion of patients with an ejection fraction >60 had mild and moderate NPDR (p-value <0.001).

**Table 15:** Correlation of severity of diabetic retinopathy with ejection fraction >60 DR: Diabetic retinopathy, HRPDR: High-risk proliferative diabetic retinopathy, PDR: Proliferative diabetic retinopathy, NPDR: Non-proliferative diabetic retinopathy, CSME: Clinically significant macular edema

			Ejection fraction >60		p value
			Yes	No	
DR severity	Mild NPDR	Frequency	16	3	<0.001
		Percentage	44.4%	4.7%	
	Moderate NPDR	Frequency	9	17	
		Percentage	25.0%	26.6%	
	Moderate NPDR with CSME	Frequency	5	11	
		Percentage	13.9%	17.2%	
	Severe NPDR	Frequency	1	7	
		Percentage	2.8%	10.9%	
	Severe NPDR with CSME	Frequency	1	7	
		Percentage	2.8%	10.9%	
	PDR	Frequency	0	4	
		Percentage	0.0%	6.3%	
	HRPDR	Frequency	0	14	
		Percentage	0.0%	21.9%	
	No DR	Frequency	4	1	
		Percentage	11.1%	1.6%	
Total		Frequency	36	64	
		Percentage	100.0%	100.0%	

## Discussion

Large retrospective studies done with significant samples of individuals with type 2 diabetes (T2D) have shown that the risk of cardiovascular disease (CVD) rises as the combined burden of microvascular disease increases [10,11]. Diabetic retinopathy (DR) is a clear sign of diabetes that is not well-controlled and is associated with an increased risk of cardiovascular disease (CVD). In addition, they have the ability to initiate inflammatory reactions that may contribute to the development of atherosclerosis, as well as causing coronary artery disease (CAD) and exacerbating cardiovascular disease (CVD) [12,13].

Diabetes mellitus and cardiovascular disease (CVD) share some of the common risk factors, such as obesity [13]. The worldwide research group from the World Health Organization (WHO) has shown that diabetic retinopathy (DR) is linked to an increased risk of cardiovascular mortality and the occurrence of myocardial infarction in patients with type 2 diabetes (T2D) [14]. The present study was undertaken to assess the prevalence and other risk factors of diabetic retinopathy among 100 IHD patients with diabetes attending an eye OPD at a tertiary care center in Western India.

Previous studies primarily focused on the incidence and risk of CVDs, including IHDs, among diabetic retinopathy patients and the subsequent outcomes in those patients. Pradeepa et al. from Chennai, India, in their large study, evaluated the correlation of DR with CAD among 1736 diabetes patients. Their cross-sectional study showed a significant association between DR and CAD in South Indian subjects with type 2 diabetes [15]. El Demerdash et al. explored the utility of DR in predicting CAD among 60 diabetes patients. They concluded that the detection of DR or increased carotid intimal thickening should be a trigger to investigate CAD, and coronary angiography has to be performed once retinopathy is diagnosed [3].

Castelblanco et al. assessed the predictive capability of DR towards CVD diseases among 374 T2DM patients in a study from the USA. They concluded that DR is a strong predictor of cardiovascular events in T2D individuals at primary CVD prevention [16]. Eid et al. from Egypt evaluated the predictive capability of DR towards coronary atherosclerosis among 140 NIDDM patients. They concluded that the presence and degree of DR are independent predictors of severe coronary atherosclerosis. Therefore, when evaluating whether a patient with T2DM is at high risk for CAD or not, the DR degree should be taken into consideration [17]. In another study from Egypt, Attia et al. determined the association of DR with the risk of CAD among 50 diabetes patients. They concluded that diabetic retinopathy is a serious risk factor for increasing the severity of coronary artery diseases and can be considered a predictor of CHD in patients with DR [18].

DR prevalence and demography

In our study, among the patients with IHD and diabetes, the prevalence of diabetic retinopathy was found to be 95%. This is high when compared with the general population rate. A study from Ethiopia reported a DR prevalence of 34.1% among diabetes mellitus patients [19]. Vashist et al., in their survey among elderly people with IHD, reported a prevalence of DR of 11.8% [20]. The variation in the DR prevalence among IHD patients in our study might be due to the fact that all included patients were also diabetics. Data from India reported a prevalence of 15.5% to 16.9% of DR among diabetes patients [20,21]. Pradeepa et al. have shown that a significant relationship exists between coronary artery disease and DR, especially among poorly controlled diabetes patients [15]. Thus, the higher prevalence of DR in our study can be attributed to the duration of diabetes and poor hypoglycemic control, as indicated by BSL values and glycated hemoglobin values among most of the patients with IHD.

In our study, the majority of the patients were in the age group of 60-69 years (46%), followed by patients in the age group 50-59 years (35%), and the mean age of patients with diabetic retinopathy and patients with no diabetic retinopathy was 58.38 and 59.40 years, respectively. Attia et al., in their study, had patients of similar age to our study (mean=59.2 years and 57.3 years) [18]. The mean age of CVD patients in a study by Castelblanco et al. was 60 years [16]. Relatively younger patients were included in the study by El Demerdash et al. (mean=46.19 years to 47.1 years) [3]. Eid et al. included DR and non-DR patients with a mean age of 57.28 years and 53.41 years, respectively [17]. Our study and the study by Attia et al. found no significant association between age and diabetic retinopathy [18]. Eid et al. found that DR patients were significantly older than patients with no diabetic retinopathy [17].

In our study, most of the patients were males (65%), while 35% were females. Eid et al. also had a higher number of male patients in their study [17]. In contrast, females were more prevalent in the Attia et al. study [18]. Castelblanco et al. also reported females as the majority of the patients with CVD events [16].

Around 63% of our patients had DM for more than five years. Poor glycemic control was noted among the majority of the patients, with 64% having a blood sugar level of 200-300 mg/dl and 21% having levels between 140 and 200 mg/dl. The mean duration of diabetes in patients with IHD in our study was significantly higher among DR patients (mean=5.97 years) than the non-DR patients (mean=3.4 years). Eid et al. reported a higher duration of DM among their DR patients (mean=7.97 years) than their non-DR patients (mean=3.54 years) [17]. Vashist et al. reported a higher risk of DR among diabetes mellitus patients who had the disease for a duration of more than 10 years and had blood sugar values above 200 mg/dl [20]. Mersha et al. also observed this in their study of Ethiopian DR patients [19].

Although a majority of the patients were on treatment with OHA (76%) in our study, 24% were on treatment with insulin. Insulin usage has also been shown to be associated with a higher incidence of DR [19,20]. We found a significant association between the severity of diabetic retinopathy and the use of insulin as a treatment modality, i.e., a higher proportion of the severe forms of DR patients were on insulin than the milder forms of the DR. Although the direct relationship may not be there, insulin intake signifies the severity and uncontrolled nature of diabetes, which in turn might have led to the DR and subsequent worsening of the DR.

In our study, HbA1c was significantly higher among the diabetic retinopathy group than the patients with no DR, which is similar to the findings of the Eid et al. study [17]. Most of the patients had HbA1c values of 5.5-7.5 (56%), followed by HbA1c values of 7.6-9.5 (30%) and >9.5 (14%). The increased severity of diabetic retinopathy with elevated glycated hemoglobin levels was correlated with the Kruskal-Wallis test. There was a significant association between the severity of diabetic retinopathy and the higher HbA1c levels in our study population. Robyn J. Tapp et al. and Afaf M. S. Al-Adsani et al. reported findings similar to our study, finding a significant association between HbA1c and the severity of diabetic retinopathy [22,23].

Relationship of DR and its severity with CVD

Research has shown that congestive heart failure (CHF) negatively affects the ability of the microvessels of the retina to expand in response to flickering light. Evaluation of the vessels of the retina has been depicted as a novel and useful method for non-invasively evaluating microvascular problems in CHF [21]. Our study shows a clinically significant relationship between the severity of DR and the severity of IHD in terms of ejection fraction.

In our study, 2D-Echo evaluation of patients with IHD showed that most of the patients had an ejection fraction of 40-60% (47%), followed by a value of >60% (36%), and a value of <40% (17%). It was observed that the patients in our study with an ejection fraction of <40 had a significantly higher severity of diabetic retinopathy in the form of PDR and HRPDR. The severity of the DR was directly correlated with the severity of IHD in our study, with most of the IHD patients with a 40-60 ejection fraction having moderate NPDR and patients with a >60 ejection fraction having mild or moderate NPDR.

Eid et al. also reported a significant positive correlation between the grade of diabetic retinopathy and the severity of the CAD among their patients. They studied the relationship between the degree of DR and the angiographic severity of CAD. Patients with advanced DR had a more severe CAD than those with mild or no DR. They concluded that when evaluating whether a patient with T2DM is at high risk for CVD, the DR severity should be taken into consideration [17]. Attia et al. reported a significantly higher risk of CAD among DR patients than non-DR patients. They reported that diabetic retinopathy was a major risk factor for enhancing coronary atherosclerosis [18]. In contrast, Castelblanco et al. reported no impact of DR severity on cardiovascular events, although DR itself had a significant relationship with the incidence of CVD events [16]. A recent comprehensive analysis revealed that diabetic retinopathy (DR) is a robust indicator of both stroke and cardiovascular disease, indicating that people with DR generally have a more unfavorable prognosis compared to those who do not have DR [24].

The majority of the patients in our study had moderate NPDR (26%), followed by mild NPDR (19%). In a study by Castelblanco et al., 45.7% of the CVD patients had moderate or severe DR. While 14% of our patients had HRPDR, none of the patients from Castelblanco et al. had a severe form of DR (neither NPDR nor PDR) [16]. Vashist et al. reported mild DR as the most common pattern of DR (11.8%) among the diabetes patients included in their study [20]. Patients with severe forms of DR (proliferative DR) had proportionately higher cases of stenosis in the coronary vessel (80%) than the NPDR patients (70%) in the El Demerdash et al. study. No such stenosis was noted in any of the non-DR patients in the same study, indicating the significant link between the CADs and the DR in the study [3].

Limitations

Several inherent limitations must be acknowledged, which may affect the generalizability and interpretation of the study findings. First, the study's single-center design, conducted at a tertiary care hospital in Western India, limits the diversity of patient demographics. Second, the cross-sectional design of the study limits the temporality of the association between DR and the other factors studied. Also, due to the lack of a control group (non-IHD patients), we could not compare the risk factors and outcomes between the two groups. Additionally, the relatively small sample size of 100 cases may limit the statistical power and robustness of the conclusions drawn. To overcome these constraints, future research should focus on prospective, multicenter studies with larger and more diverse patient populations.

## Conclusions

The prevalence of diabetic retinopathy among the IHD patients with diabetes was 95% in our study, with moderate NPDR being the most common stage of DR seen among the patients. It was observed that patients from rural backgrounds and illiterate people had a greater prevalence of diabetic retinopathy. More severe stages of retinopathy were seen in these patients. The duration of IHD and DM had a positive association with the presence of diabetic retinopathy.

It was observed that more severe stages of diabetic retinopathy were seen in patients who were on treatment with insulin than in patients who were on treatment with OHA. Severe stages of diabetic retinopathy were associated with higher BSL (R) levels and higher glycated hemoglobin levels. In the present study, it was observed that a lower ejection fraction (<40), which is a marker of reduced cardiac function, was associated with more severe stages of diabetic retinopathy. Patients with a higher ejection fraction (>60), which indicates good cardiac function, were associated with milder stages of diabetic retinopathy. Further, multi-centric studies need to be undertaken to validate and improve the generalizability of our findings.
